# Supplementary material for: Safflower Polysaccharides Alleviate TNBS-Induced Colitis by Modulating Gut Immunity
Source: Foods. 2025 Sep 14;14(18):3199. doi: 10.3390/foods14183199 (PMC12469953; doi:10.3390/foods14183199)
Supplement: Supplementary file 1 [file foods-14-03199-s001.zip › foods-3793274-supplementary.pdf]

Supplementary Figures

Safflower Polysaccharides Alleviate TNBS-Induced Colitis by Modulating Gut Immunity

Chao Jiang, Furong Zhu, Shabaaiti Aimaier, Liang Zhang, Md Hasan Ali, Furong Fan, Yating Lu, Mengwei Jia, Dongsen Wu, Haipeng Yin, Jianwang Wei, Shenghui Chu * and Min Liu *

Key Laboratory of Xinjiang Phytomedicine Resource and Utilisation, Ministry of Education, Institute for Safflower Industry Research, Pharmacy College, Collaborative Innovation Center for Efficient Safflower Production and Resource Utilization of XPCC, Shihezi University, Shihezi 832002, China; 13918049521@163.com (C.J.); zfr210921@163.com (F.Z.); sabaat_hven@163.com (S.A.); zhangliang@stu.shzu.edu.cn (L.Z.); hasan.yzu@gmail.com (M.H.A.); f721572869@163.com (F.F.); 15979116392@163.com (Y.L.); jmw079@126.com (M.J.); wudongsen@stu.shzu.edu.cn (D.W.); 13565162388@163.com (H.Y.); w16650554611@163.com (J.W.)

* Correspondence: chushenghui2022@163.com (S.C.); liuminshzu@163.com (M.L.)

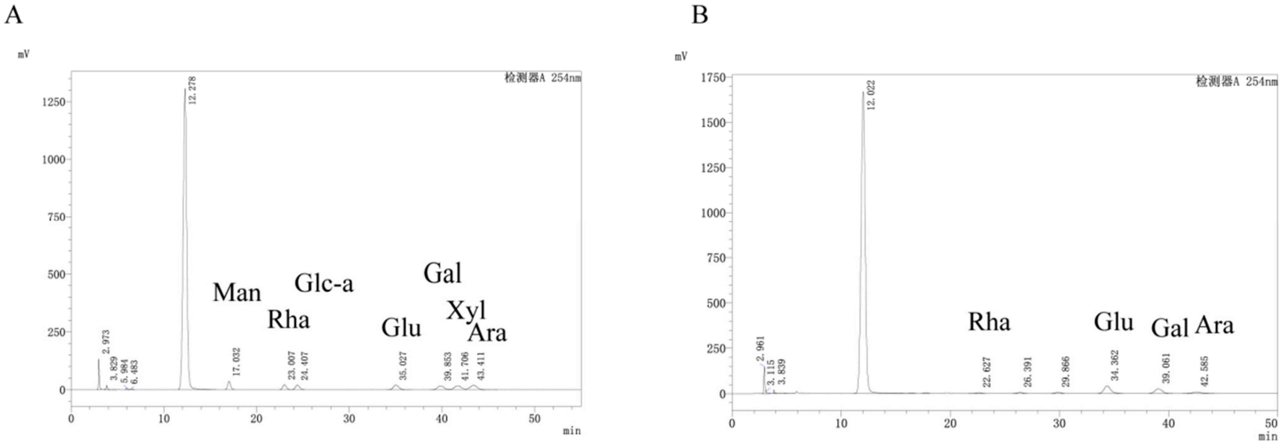

Supplementary Figure S1. The HPLC chromatogram of Monosaccharide standards(A) and The HPLC chromatogram of Monosaccharide composition of SPS.

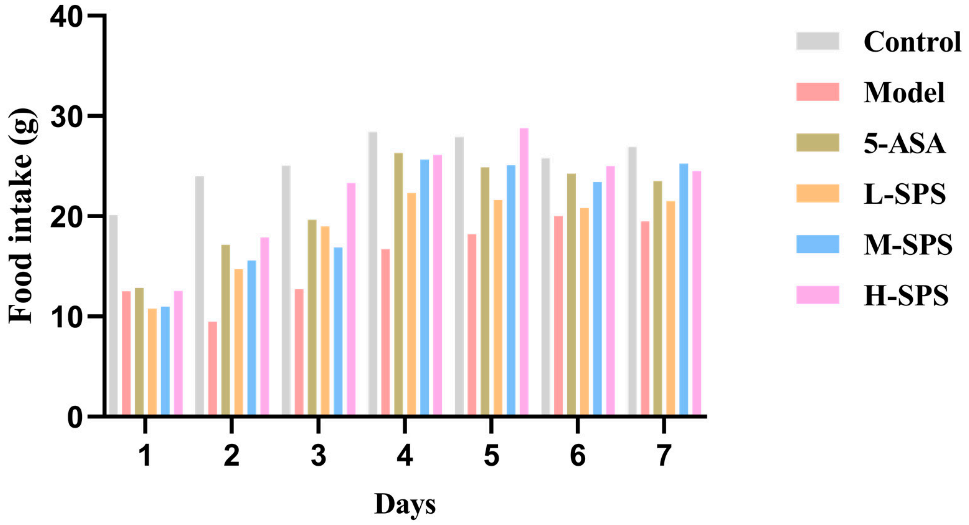

Supplementary Figure S2. Effect of SPS on food intake in UC rats.

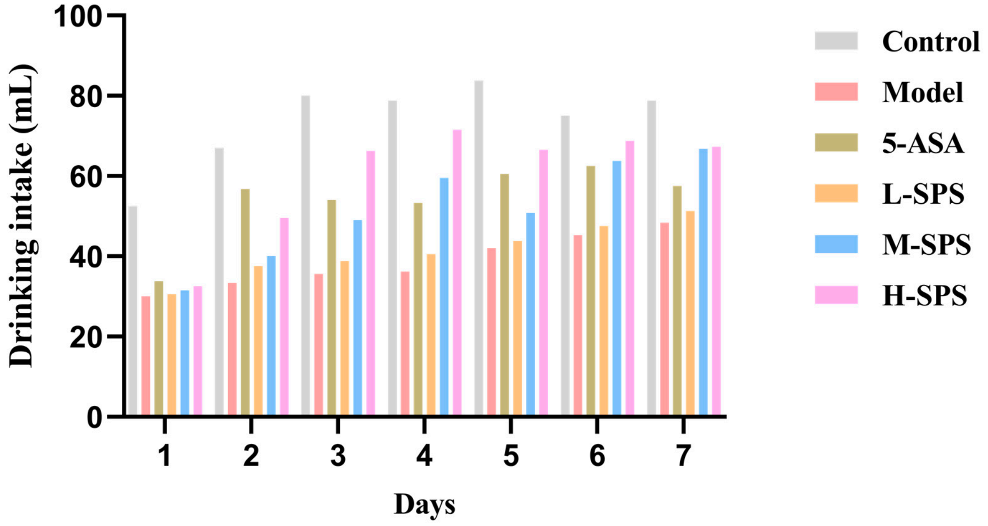

Supplementary Figure S3. Effect of SPS on drinking intake in UC rats.

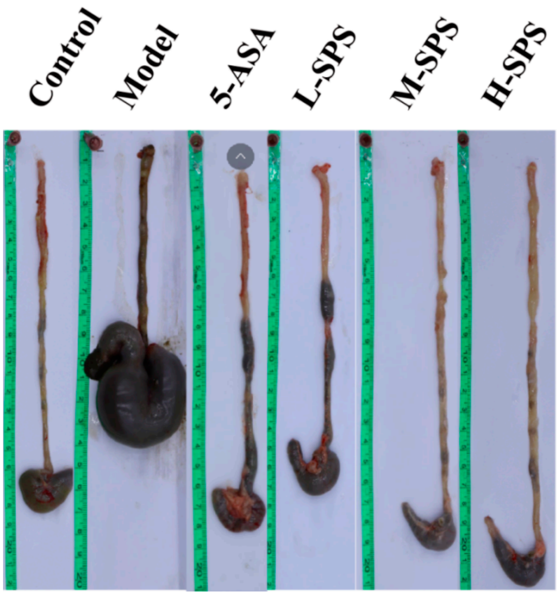

Supplementary Figure S4. Colon representation figure.

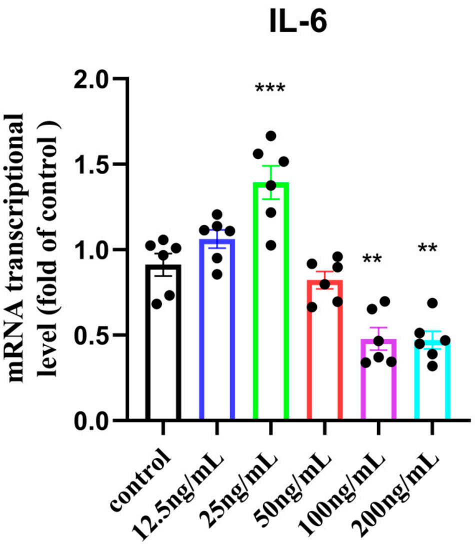

Supplementary Figure S5. The effect of different concentrations of TNF on the secretion of pro-inflammatory factors in Caco-2 cells.The data are presented as the mean ± SEM, n = 6. * P < 0.05 and *** P < 0.001 versus the control group.

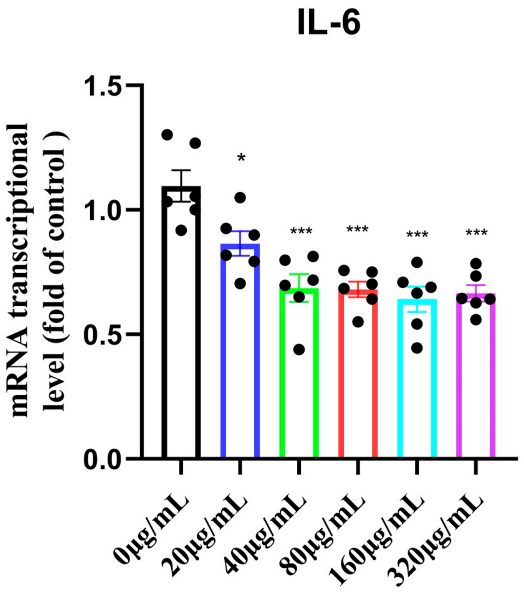

Supplementary Figure S6. The screening of optimal intervention concentration for SPS.The data are presented as the mean ± SEM, n = 6. ** P < 0.01 and *** P < 0.001 versus the control group.

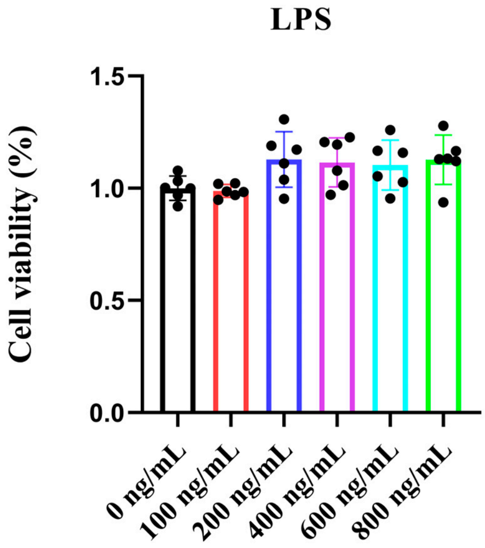

Supplementary Figure S7. The cytotoxic impact of LPS onTHP-1 cells.

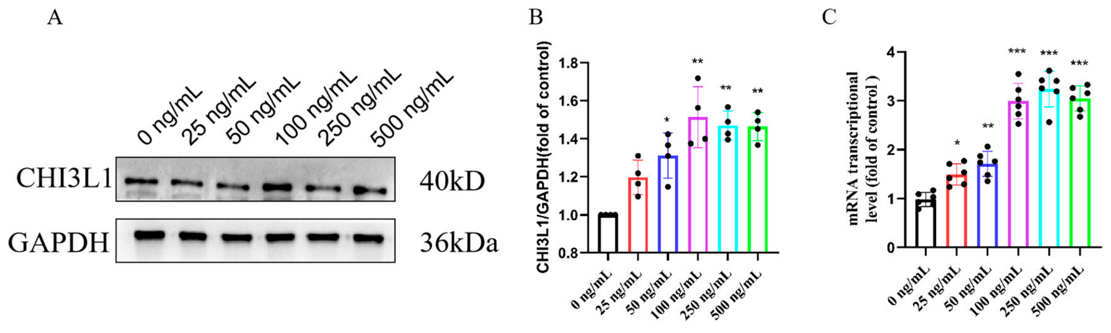

Supplementary Figure S8. Screening of optimal conditions for CHI3L1 overexpression. (A) Protein expression level of CHI3L1. (B)CHI3L1 relative protein expression statistics.(C)The mRNA levels of CHI3L1.The data are presented as the mean ± SEM, n ≥ 4. ** P < 0.01 and *** P < 0.001 versus the control group.

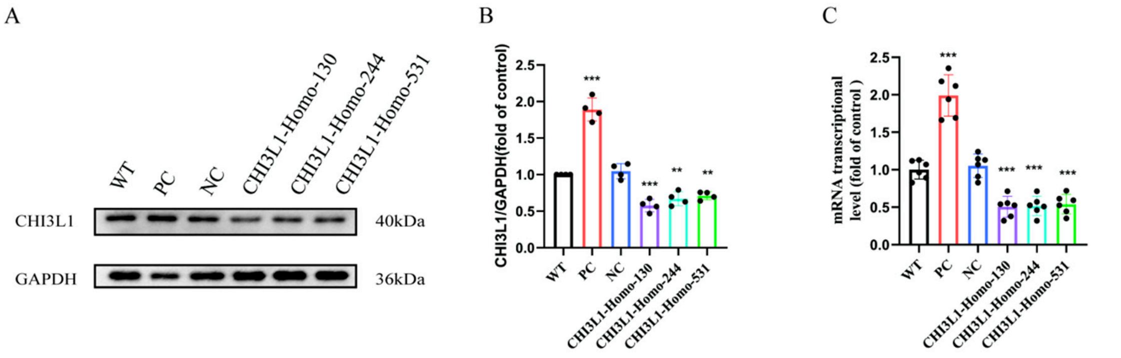

Supplementary Figure S9. Screening of optimal conditions for CHI3L1 knockdown. (A) Protein expression level of CHI3L1. (B)CHI3L1 relative protein expression statistics.(C)The mRNA levels of CHI3L1. The data are presented as the mean ± SEM, n ≥ 4. ** P < 0.01 and *** P < 0.001 versus the control group.

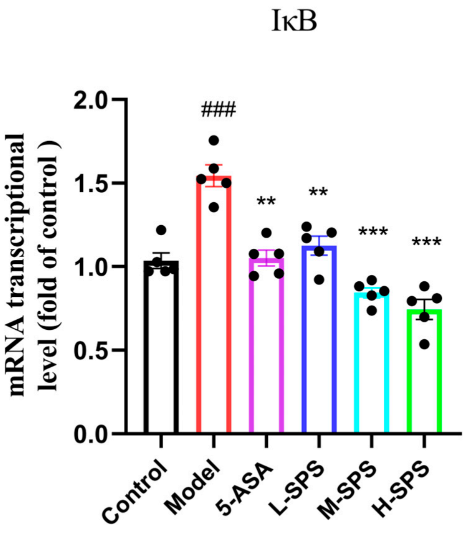

Supplementary Figure S10. The mRNA expression levels of IκB in rats .The data are presented as the mean ± SEM, n = 5. ### P < 0.001 versus the Control group. ** P < 0.01 and *** P < 0.001 versus the Model group.

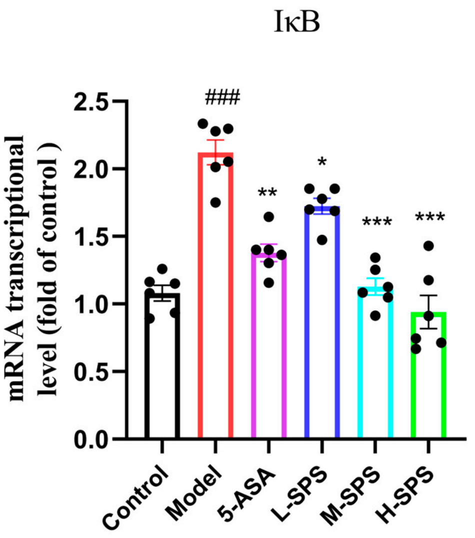

Supplementary Figure S11. The mRNA expression levels of IκB in epithelial cells .The data are presented as the mean ± SEM, n = 6. ### P < 0.001 versus the Control group. * P < 0.05, ** P < 0.01 and *** P < 0.001 versus the Model group.

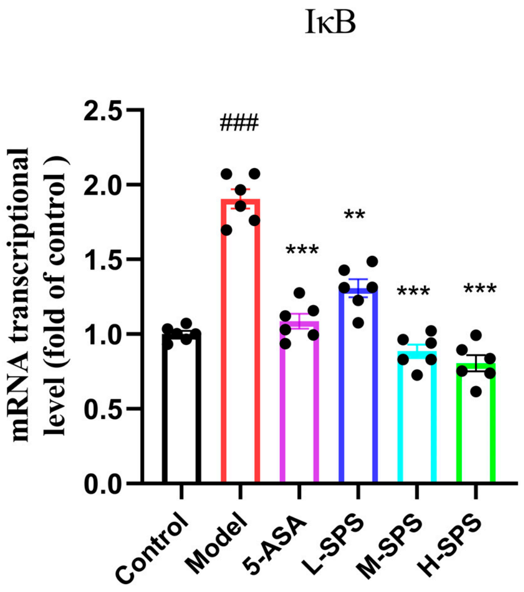

Supplementary Figure S12. The mRNA expression levels of IκB in THP-1 cells .The data are presented as the mean ± SEM, n = 6. ### $P < 0.001$ versus the Control group. ** $P < 0.01$ and *** $P < 0.001$ versus the Model grou

Supplementary Table S1 . Parameter of SPS.

	Indicator	Content
Composition (%)	Total sugar	60.32
	Protein	2.89
	Polyphenols	1.26
Monosaccharides (%)	Glucose	21.63
	Galactose	15.60
	Rhamnose	2.47
	Arabinose	5.50
Average molecular weight (kDa)		31.11
